# Insights Into the Role of Mitochondria in Vascular Calcification

**DOI:** 10.3389/fcvm.2022.879752

**Published:** 2022-04-29

**Authors:** ZL Zeng, Qing Yuan, Xuyu Zu, Jianghua Liu

**Affiliations:** ^1^Department of Metabolism and Endocrinology, The First Affiliated Hospital, Hengyang Medical School, University of South China, Hengyang, China; ^2^Department of Clinical Medicine, The First Affiliated Hospital, Hengyang Medical School, University of South China, Hengyang, China; ^3^Key Laboratory for Arteriosclerology of Hunan Province, Department of Cardiovascular Disease, Hengyang Medical School, University of South China, Hengyang, China

**Keywords:** vascular calcification, mitochondria, oxidative stress, extracellular vesicles (EVs), atherosclerosis

## Abstract

Vascular calcification (VC) is a growing burden in aging societies worldwide, and with a significant increase in all-cause mortality and atherosclerotic plaque rupture, it is frequently found in patients with aging, diabetes, atherosclerosis, or chronic kidney disease. However, the mechanism of VC is still not yet fully understood, and there are still no effective therapies for VC. Regarding energy metabolism factories, mitochondria play a crucial role in maintaining vascular physiology. Discoveries in past decades signifying the role of mitochondrial homeostasis in normal physiology and pathological conditions led to tremendous advances in the field of VC. Therapies targeting basic mitochondrial processes, such as energy metabolism, damage in mitochondrial DNA, or free-radical generation, hold great promise. The remarkably unexplored field of the mitochondrial process has the potential to shed light on several VC-related diseases. This review focuses on current knowledge of mitochondrial dysfunction, dynamics anomalies, oxidative stress, and how it may relate to VC onset and progression and discusses the main challenges and prerequisites for their therapeutic applications.

## Introduction

Vascular calcification (VC) is prevalent in diabetes mellitus, atherosclerosis, and chronic kidney disease (CKD), is increasing progressively during aging, and is closely associated with many cardiovascular events and mortality (1, 2). Scientists have long struggled to explain the mechanism of VC, although significant progress has been made, we have not yet fully understood VC etiology and pathogenesis, but one thing confirmed is that mitochondrial homeostasis, including mitochondrial biogenesis, mitochondrial dynamics, glucose and fatty acid metabolism, oxidative stress, autophagy, and apoptosis, has been shown to be extensively involved in the onset and development of VC.

Mitochondria are the center of energy metabolism in eukaryotic cells, participating in a variety of metabolic biochemical activities, including oxidative phosphorylation (OXPHOS), the Krebs cycle, fatty acid-oxidation, calcium management, and heme biosynthesis (3). Moreover, it has recently been shown that mitochondria can no longer be considered as simple bioenergetics factories but rather as platforms for intracellular signaling and regulators for many biological processes (4). Meanwhile, these organelles are highly dynamic organelles that undergo constant fusion, fission, and degradation (5); damage of any step in this process will cause mitochondrial dysfunction (MFD). As a result, MFD has been associated with many cardiovascular diseases, as well as VC (6).

Mitochondria contain multiple electron transporters that can form a broad network of producing reactive oxygen species (ROS) and antioxidant defenses. Multiple insults, including oxidative damage itself, can cause an imbalance between production and detoxification of ROS and cause oxidative damage to biomolecules, including mitochondrial DNA (mtDNA), and then induce VC (7). In this study, we reviewed how mitochondria participate in VC and how these insights may usher in a new era of mitochondrial-targeted therapies.

## Overview of VC Mechanisms

Vascular calcification is the process of the active deposition of calcium phosphate on the blood vessel wall under the cell-mediated process, which can be summarized in three stages as follows: (1) pro-calcification factors induce vascular smooth muscle cell (VSMC) osteogenic differentiation; (2) differentiation VSMCs release matrix vesicles; (3) matrix vesicles promote the formation of hydroxyapatite and are deposited on the blood vessel wall, ultimately promoting the onset of VC. In this study, we briefly summarized the classification and multiple mechanisms of VC.

### VC Classification

**Figure 1:**
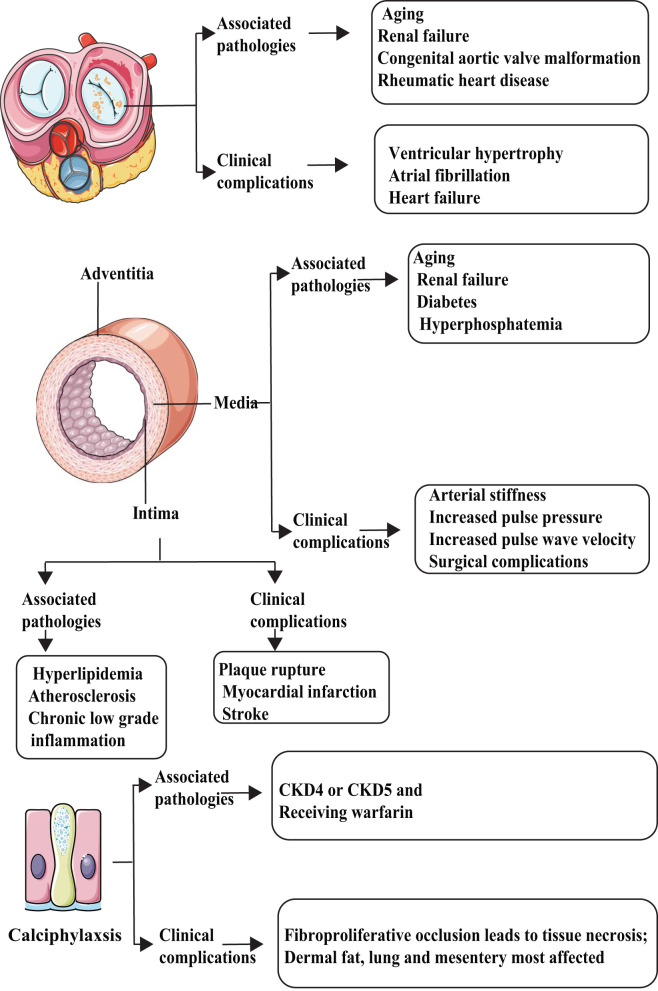
Types and characteristics of vascular calcification (VC). VC can be divided into at least five categories: intimal, media, adventitia, valvular calcification (ValvC), and calciphylaxis. They have different inducer and clinical complications.

Using microanatomic and clinical criteria, at least five types of VC can be identified (Figure 1). According to the location where VC occurs and the pathophysiological mechanisms, VC can be divided into intimal, medial, adventitia, valvular calcification (ValvC), and calciphylaxis; from another perspective, based on the calcification size, VC can be divided into microcalcification and macrocalcification (8). The definition of VC *via* size is unclear; it is generally believed that microcalcification refers to punctate calcifications with a diameter of <15 μm. Almost all microcalcifications can be seen in the thickened intima (9).

The main factors that promote intimal calcification, including lipid metabolism abnormalities and chronic inflammation, may begin with VSMC apoptosis and release of matrix vesicles, which mainly lead to arterial obstruction and plaque rupture. Most abundant calcification exists in fibrocalcific plaques followed by healed ruptured plaque. Conversely, erosion and pathological intimal thickening are less commonly found (9). In contrast, the main factors that promote medial calcification include diabetes/metabolic syndrome (MetS) and CKD, which are linked to increased arterial stiffness, systolic hypertension, and increased pulse wave velocity, leading to increased diastolic dysfunction and heart failure (10).

Up to date, few studies aim at adventitia-related calcification. Traditionally, the primary function of adventitia was considered to sustain vessels, give nutrition to the intima, and maintain sympathetic nerve terminals. However, some studies suggest that the adventitia also plays an important role in atherosclerosis development through inflammation response (11, 12). Song et al. (13) demonstrated the presence of adventitia calcification in ApoE^−/−^ mice (a commonly used mouse model of AS and VC); however, the specific mechanisms governing adventitia calcification are not clear. By far, ValvC is considered an aging-related degeneration disease. ValvC will lead to ventricular hypertrophy, atrial fibrillation, and heart failure (14).

Calciphylaxis is a rare, life-threatening syndrome of VC related to end-stage renal disease and warfarin therapy, characterized by occlusion of microvessels within the subcutaneous adipose tissue and dermis that leads to intense pain and ischemic skin lesions. Once calciphylaxis has been diagnosed, the prognosis is typically poor (survival, <1 year) (15, 16). For more knowledge on this broad scientific area, readers are referred to some exhaustive reviews (17, 18).

### Diverse Cell Types Involved in VC

**Figure 2:**
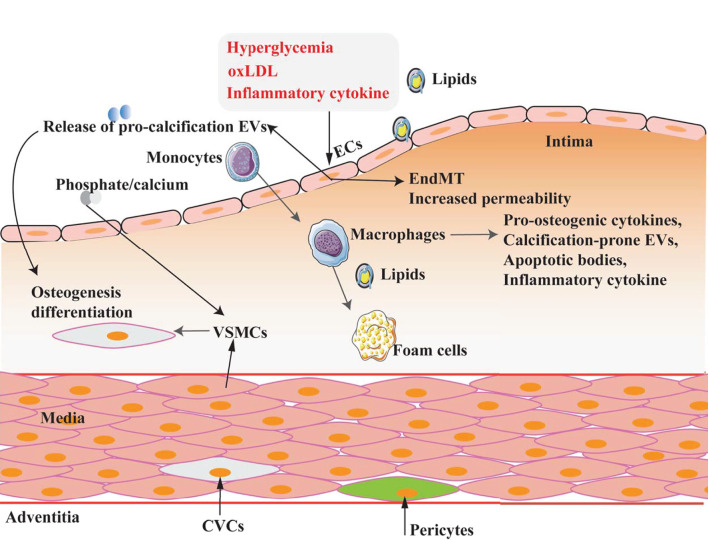
Multicell types involved in VC. VC is a complex process in which multiple cell types are involved and play different roles under different pathophysiological conditions.

Vascular calcification is a complex pathological process involving multiple cell types, including VSMCs, vascular endothelial cells (VECs), pericytes, and macrophages (19). They play various roles in the different pathological stages of VC, as summarized in Figure 2.

#### Vascular Smooth Muscle Cells

Vascular smooth muscle cells are the primary cell type of arterial wall, which help maintain the elasticity and contractility of blood vessels. The contractile phenotype is the primary function of normal VSMCs and exhibits low proliferative and synthetic characteristics. Although VSMCs are terminally differentiated cells, they exhibit a highly plastic phenotype (20). Under pathological conditions, VSMCs can transform into various phenotypes, including osteogenic, adipose, and macrophage phenotypes (21).

**Figure 3:**
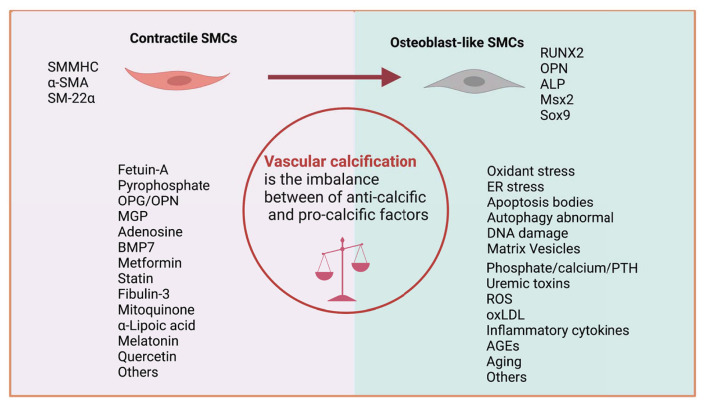
Imbalance between anti-calcific and pro-calcific factors. VC can be recognized as the imbalance of calcification inducers and inhibitors. During this process, VSMC marker (SMMHC, α-SMA, and SM-22α) expression is downregulated, and calcification marker (RUNX2, ALP, MSX2, and SOX9) expression is upregulated.

Numerous factors have been identified as contributors to the VSMC osteogenic phenotype transformation, which can be considered as an imbalance between anti-calcific (e.g., Fetuin-A, pyrophosphate, MGP, BMP7, and OPG/OPN) and pro-calcific (e.g., phosphate/calcium, PTH, uremic toxins, oxLDL, AGEs, and inflammatory cytokines) (Figure 3) (22). In the process of VC, VSMCs transform from the normal contractile phenotype to osteogenic (also called synthetic phenotype) that exhibits higher growth and migration rates and expresses markers of the osteoblast or chondroblast phenotypes such as RUNX2, ALP, and fewer VSMC markers such as α-SMA and SM-22α (8).

#### Vascular Endothelial Cells

Vascular endothelial cells are the primary cell type in the vascular intima and build the barrier between blood and vascular wall to prevent or reduce the damage caused by harmful substances in the blood flow and play an essential role in the structural and functional integrity of arteries (23). VEC dysfunction is not only involved in atherosclerosis but also plays a crucial role in the process of VC (24, 25). Nowadays, the mechanism related to VECs in VC is mostly focusing on endothelial-mesenchymal transition (EndMT), angiogenesis, the release of pro-calcification signaling molecules, and EVs.

Endothelial-mesenchymal transition is the process of losing endothelial features and acquiring mesenchymal or myofibroblastic phenotypes eventually leading to cells with osteogenic potential and playing pivotal roles in VC (26). Deficiency of matrix Gla protein (MGP), a bone morphogenetic protein (BMP)-inhibitor, promotes EndMT of VECs, which are differentiated into osteoprogenitor cells, and finally induces VC (27). High glucose (HG) also caused cardiovascular calcification through BMP signal activation-induced EndMT (28).

Inflammatory cytokines act as major effectors of the signal pathway to regulate VC. Inflammatory cytokines including tumor necrosis factor-alpha (TNF-α) and interleukin-1 beta (IL-1β) can promote VC through induced EndMT *via* BMPR2-JNK signaling pathway (29). Calciprotein particles are calcium phosphate-containing nano-aggregates that contribute to VC by inducing oxidative stress and the upregulation and release of TNF-α (30). Moreover, the inflammatory signaling pathway block is a potential therapeutic strategy for VC. For example, 6-Shogaol (one of the Gingerols and others include 4-, 8-, 10-, and 12) (31) can reduce VC *via* inhibiting Akt/NLRP3/IL-1 signaling (32). Similarly, caffeic acid phenethyl ester mitigates aortic valve interstitial cell calcification through block Akt/NF-κB/NLRP3 inflammasome pathway (33).

The earliest evidence that angiogenesis and ectopic calcification may be associated was revealed in the 1980s (34). Since then, the bulk of evidence supporting angiogenesis in atherosclerotic VC has been obtained from histological investigations that used endothelial cell markers (e.g., von Willebrand factor and CD31) to detect new blood vessels inside the arterial wall. The molecular mechanisms of angiogenesis in VC remain poorly understood. Possible mechanisms included were as follows: first, the invading blood vessels have the potential to serve as a conduit for osteoprogenitor cells (35); second, some angiogenic factors such as vascular endothelial growth factor can also affect osteogenic and chondrogenic cells (36); and finally, vasculature can act as a catalyst for inflammation and boost VC (37, 38).

In addition to direct differentiation to osteoblast-like cells, VECs also mediate VC by releasing calcification potential EVs under pathological conditions. The released EVs from HG stimulated VECs can induce VSMC aging and calcification through the mTOR signaling pathway (39).

#### Macrophages

Macrophages have strong plasticity and functional heterogeneity. The primary two subsets are “the classical activated macrophage” (M1) and “the alternative activated macrophage” (M2) (40). Through releasing pro-osteogenic cytokines, calcification-prone EVs, and apoptotic bodies, macrophage plays a vital role in VC (41).

Molecular imaging use co-injected with fluorescent nanoparticles to visualize macrophages (NIRF nanoparticle) and calcification (OsteoSense) study reveals macrophage accumulated in calcification area (42). Moreover, macrophages usually enriched in vulnerable plaques have a more significant calcification burden, especially in patients with diabetes due to the release of pro-inflammatory factors such as IL-1β and TNF-α (43, 44).

Basic calcium phosphate (BCP) crystals consist mainly of calcium hydroxyapatite (45). BCP stimulates macrophage secretion of TNF-α through PKC, ERK1/2, and JNK signaling and promotes VC (46). Furthermore, Runx2 (an essential regulator of VC) directly binds to the promoter and upregulates the expression of receptor activator of nuclear factor-B ligand (RANKL). Additionally, RANKL promotes osteoclastogenesis and migration of macrophages during VSMCs calcification (47).

#### Pericytes

Pericytes are perivascular cells of mesenchymal origin found in all vascularized organs with multiple functions including vessel growth, permeability, and contractility (48). To date, the knowledge of pericyte is still very limited due to the lack of truly specific markers (49).

Pericytes are characterized as irregular cells with ruffled edges, lack of migration into 3-D collagen gels, the existence of α-SMA, immunoreactivity with mAb 3G5, and the absence of immunoreactivity with antibodies against von Willebrand factor (vwf) were all observed (50, 51). Pericytes involved in regulating angiogenesis and pericytes as progenitor cells have the potential differentiation to osteoblasts and chondrocytes directly, so involved in the VC process (52).

Pericyte infiltration correlates positively with VC; one hypothesis is that pericytes may be resident vascular progenitor cells found in the adventitia that have the potential to move into the media and intima of the arteries, affecting surrounding cells, and differentiate into osteoblasts when subjected to pathological stress. The RANK/RANKL/OPG signaling pathway is currently considered to be the main pathway for pericyte-mediated VC (53). Glucocorticoids are synthetic steroid that has anti-inflammatory and immunosuppressant effects. Studies reported that glucocorticoid-dexamethasone promotes pericyte osteogenic differentiation and downregulates genes associated with inhibition of mineralization, including matrix Gla protein (MGP), osteopontin (OPN), and VC-associated factor (VCAF) (54). However, studies on the role of pericytes in VC are limited and need to be investigated further.

### Bone-Vascular Axis

**Figure 4:**
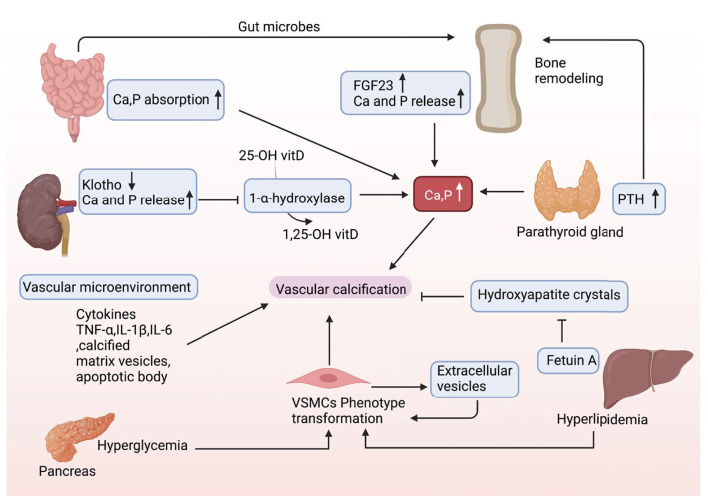
Organs cross talk in VC. During the onset and development of VC, there may be multiple factors interacting with each other and cross talk between different organs.

Organ interactions are involved in VC (Figure 4). Bone-vascular axis is one of the typical representatives. After the skeleton, the vasculature is the second most calcified structure in human. The vasculature provides the sustentacular niche for the development of osteoblast progenitors and is the conduit for the egress of bone marrow cell products arising, in turn, from the osteoblast-dependent hematopoietic niche (55). Furthermore, osteoporosis is usually accompanied by VC, and there is a positive association between the degree of aortic calcification and bone loss (56). Notably, numerous bone matrix proteins are expressed in calcified arteries, suggesting the presence of a bone-vascular axis (57).

Osteoblast-like cells are absent in the normal arterial wall. However, in a VC-induced context, the VSMCs can differentiate into chondroblast/osteoblast-like cells (17). VC shares with osteoporosis similar risk factors, such as smoking, sedentary lifestyle, and dyslipidemia (58). Calcium salts released from bone can deposit in the vascular lesions and the vascular systems, and bone shows considerable anatomical parallelism and functional cross talk, which further participate in VC occurrence (59–61). Whether the relationship between VC and osteoporosis is causal or parallel? This is an interesting research direction.

### Reaction to Vascular Injury Hypothesis

“Vascular injury response theory” is one of the critical hypotheses of AS; more importantly, endothelial injury can not only trigger the process of AS but also initiate intimal and medial calcification by releasing apoptotic bodies and matrix vesicles containing cell debris (24). After vascular injury, VSMCs transform from contractile phenotype to synthetic phenotype, migrate to the site of vascular injury, and proliferate and secrete matrix and other components to form a fibrous cap, thus isolating lipid rich and procoagulant plaque core from blood. Usually, after this process, the VSMCs will return to the contractile phenotype, but if the injury stimulation persists, the VSMCs may convert to the phenotype of osteoblast, cartilage, or adipocyte, and start the process of calcification (62). Moreover, chronic inflammation and immune response after vascular injury may also participate in the regulation of VC (63).

### Shear Stress

Laminar flow has a vascular protective effect whereas disturbed flow exerts a harmful effect. The development of VC is also tightly regulated by mechanical stress especially on the endothelial surface (shear stress); the evidence from VC prefers to occur at branched or bifurcated areas of the vasculature, and these regions are mainly characterized by shear stress that will induce the damage of VECs; in contrast, laminar flow upregulation KLF2 and inhibition of endothelial BMP/SMAD1/5 signaling alleviate VC (64).

When hydroxyapatite deposits on the thin fibrous cap, the local shear stress can increase by 47.5%, which significantly increases the risk of plaque rupture (65). Coronary flow differences between left coronary cusp case, right coronary cusp case, and non-coronary cusp case show significant impact on leaflet kinematics and sinus flow hemodynamics. The left coronary cusp has a higher likelihood of calcification compared with the right (66). Corresponding laminar shear stress physiological status activates the cAMP/PKA pathway, and this pathway downregulates the expression of BMP-4 in the vascular endothelium contributes to the anti-VC effect (67).

### Extracellular Vesicles in VC

Extracellular vehicles (EVs) are nano-sized lipid-bound vesicles that are released from cells into the extracellular space (68). Ultrastructural analyses have identified the presence of EVs in calcified human aortic valves and medial arterial calcifications (69). In the normal environment, vascular cells release EVs that serve a physiological function and preserve homeostasis, but in a pathological environment due to changes in cytoskeletal orientation or impairment in vesicle trafficking and cargo loading, certain vesicles acquire properties that promote calcification potential (70). Recent years, many studies explored the contributions of EVs in VC. VSMC-mediated calcification involves the release of calcifying EVs, a subpopulation traditionally known as matrix vesicles (71). Macrophage-derived EVs contribute to the development of microcalcification in atherosclerotic plaques (72).

Using three-dimensional collagen hydrogels that mimic structural features of the atherosclerotic fibrous cap and high-resolution microscopic and spectroscopic analyses, Joshua et al. (73) demonstrated EVs derived from VSMCs which cultured in calcifying media promote microcalcification and affect the stability of atherosclerotic plaques. Another study demonstrates sortilin that promotes VC *via* its trafficking function of tissue non-specific alkaline phosphatase (TNAP) to EVs that lead to high mineralization competence in the extracellular milieu (74). Matrix vesicles derived from CKD rats can promote VC through increased MEK1 and ERK signaling and enhance intracellular calcium from sarcoplasmic reticulum stress (75).

Recently, it has been found that some common substances with anti-VC effects, such as melatonin, and its effects may be related to the induction of VSMCs to release exosomes with anti-VC effects (76). Moreover, bone marrow mesenchymal stem cell exosomes can suppress phosphate-induced VC *via* SIRT6–HMGB1 deacetylation (77). As an important mediator of intercellular communication, EVs, especially exosomes, have a crucial role in disease development, including VC, and hold great potential as a diagnostic marker and therapeutic targets. In addition, EVs can serve as targeted delivery vehicles for drugs and can improve bioavailability.

### Arterial Calcification and Plaque Stability

Biomechanical changes not only promote the formation of calcification but also have a significant impact on the stability of AS plaque. The difference of biological stress between calcification and soft tissue leads to the change of biomechanics, which will affect the stability of atherosclerotic plaque (78). Finite-element modeling of the stress distribution within atherosclerotic plaques has shown that microcalcifications in the fibrous cap may promote instability of the plaque, but that macrocalcifications can stabilize it, and the calcification morphology and the plaque's collagen content are the crucial factors that impact plaque stability (73).

## Mitochondria in VC

**Figure 5:**
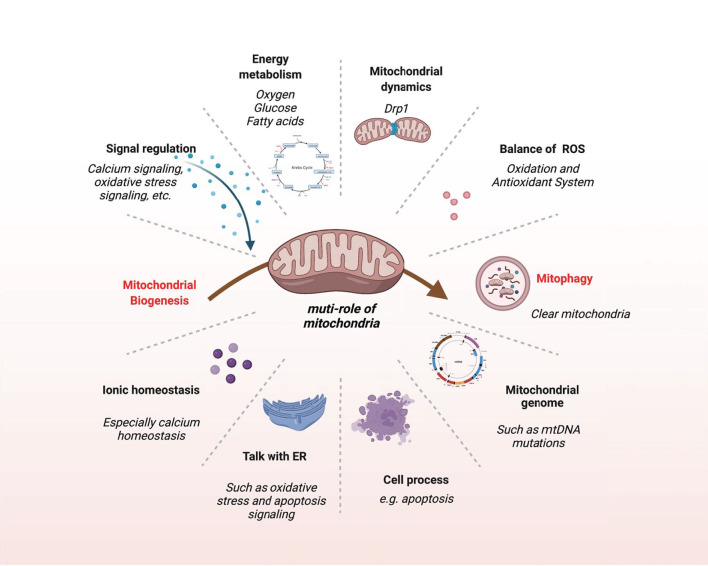
The multifaceted role of mitochondria. This schematic shows the main biological events of mitochondria.

Mitochondria are not only the center of energy metabolism but also essential organelles involved in many biological events. Any abnormality in mitochondria will affect the onset and development of VC (Figure 5).

Human arteries are mainly consisting of three layers. The intima, which is in direct contact with blood flow, is mainly covered by VECs, and the main cell type in the medial layer is VSMCs; the adventitia consists of fibroblasts, vasa vasorum, nerve endings, and a few resident inflammatory cells, and the blood vessels are covered by fatty tissues (79). The main function of the vascular system is to transport oxygen, nutrients, and metabolites throughout the body.

Mitochondria are one of the key factors to keep the structural and functional integrity of the vascular system, and mice lacking proteins required for mitochondrial activity often perish at the same developmental stage as the cardiovascular system (80). Various pro-calcification factors lead to changes in mitochondrial structure, dynamics, and function, which can lead to autophagy dysfunction, apoptosis, and activation of calcification signaling pathways, ultimately aggravating VC (Figure 5).

### Mitochondrial Content

Mitochondrial homeostasis, a dynamic process regulated by complex and coordinated biochemical signals, is mainly being held by the balance between mitochondrial biogenesis and mitophagy (mitochondrial removal) (81–83). As the central organelle of eukaryotic energy metabolism, mitochondria play a crucial role in VC. The mitochondria generally account for 3–5% of the volume of VSMCs (84) and 2–6% in ECs (85). Mitochondrial biogenesis is a complicated and poorly understood process that involves the replication of mtDNA and the expression of nuclear and mitochondrial genes; defects in mtDNA cause mitochondrial malfunction, which promotes inflammation, apoptosis, and cell senescence in vascular cells (86).

Mitochondria are highly dynamic organelles that continuously fusion and fission according to actual needs; for example, moderate exercise can enhance mitochondrial biogenesis (87). New mitochondria are synthesized from the growth and fission of preexisting mitochondria and are mainly regulated by the peroxisome proliferation-activated receptor γ coactivator 1α (PGC-1α); nuclear respiration factors NRF1 and NRF2 control the expression of the nuclear genome-encoded electron transfer chain subunits and bind to the promoters of genes involved in mtDNA transcription (88, 89).

Aging and damaged mitochondria are mainly removed by mitophagy (90). Mitophagy exerts protective effects in response to cellular stress in the cardiovascular system. Mitophagy is activated during stress to maintain the mitochondrial quality and quantity by removing damaged or superfluous mitochondria (91). β-GP treatment significantly reduces VSMCs mitochondrial biogenesis via activation of PDK4, which is reflected by the decrease of mitochondrial DNA copy number. Moreover, β-GP also increases oxidative stress, apoptosis and promotes the osteogenic differentiation of VSMCs, whereas metformin reduces β-GP-induced PDK4 expression and inhibits the osteogenic differentiation of VSMCs. Meanwhile, this research also showed that mitophagy is close related to mitochondrial biogenesis in VSMCs. Although metformin can reduce β-GP-induced PDK4 expression and inhibits its effects including oxidative stress and ROS generation, moreover, the research indicates that reduced inhibits of mitophagy can decrease mitochondrial biogenesis (92). The imbalance between mitochondrial biogenesis and mitophagy is an important pro-calcification factor.

### Mitochondrial Calcium Homeostasis

Calcium is a critical second messenger involved in intracellular and extracellular signaling cascades and is required for vascular physiology; more importantly, studies now recognize that calcium can participate in vascular pathophysiology by interacting with ROS to regulate a series of signaling pathways (93).

Calcium phosphate crystals deposited into either the medial or intimal layers of the vasculature are a critical factor for VC, and mitochondria are critical regulators of intracellular Ca2^+^ and Pi levels (94). The handle of calcium homeostasis is one of its most fascinating aspects, and the early discovery of how cells load calcium into matrix vesicles (MVs) responsible for VC is based on the observation that copious mitochondrial calcium is transported to MVs (95). The maintenance of calcium and phosphorus homeostasis in physiological states depends on a variety of uptake and efflux mechanisms, but overload ions will concentrate in mitochondria and form Ca–P complexes, therefore beginning the VC process (96). Moreover, mitochondrial calcium overload is closely associated with mitochondria ROS (mtROS) levels; mitochondrial Ca2^+^ accumulation promotes mitochondrial metabolism, activates nitric oxide synthase, and inhibits complex IV enhancing mtROS generation (97). The production of mtROS is tightly influenced by the open ratio of mPTP. The opening of mPTP results in the rapid collapse of ΔΨm and membrane depolarization, resulting in increased mtROS; and oxidative stress, in turn, stimulates mitochondrial Ca2^+^ overload *via* mPTP (96, 98).

To summarize, there is still a lack of clinical evidence and definitive mechanisms of action to demonstrate the clear role of mitochondrial calcium homeostasis in VC. Ca2^+^ under normal physiological conditions activates the mitochondrial metabolism to protect cells from calcium-induced cytotoxicity; however, dysregulated may lead to oxidative stress, inflammation, and cell death, contributing to VC (99). Therefore, exploring mechanisms regulating mitochondrial Ca2^+^ and its role in the ectopic deposits in the vascular wall may allow the discovery of novel therapeutic agents in VC.

### Mitochondrial Epigenetics and mtDNA Damage in VC

Human mtDNA is a circular double-stranded DNA molecule with a length of 16,569 bp containing a heavy (H) and a light (L) strand. The 16.6 kb genome has less intergenic spacing and encodes 37 genes (13 protein-coding genes), all involved in oxidative phosphorylation; 2 rRNAs; and 22 tRNAs (100). In contrast to nuclear DNA, mtDNA is intron less, is maternally inherited, lacks protecting histones, and possesses less-robust DNA repair mechanisms (101).

Despite mitochondrial epigenetics being a fascinating area of research, the role of epigenetics in mitochondria still remains a controversial notion. Recent findings, however, indicate that VC is related to abnormal mtDNA methylation (102). Presumably, CpG mtDNA methylation is carried out by mtDNA methyltransferase (mtDNMT1), an isoform of the maintenance methylase DNMT1 that contains a mitochondrial targeting sequence, which plays a critical role during mitochondrial malfunction and oxidative stress (103, 104). Furthermore, previous study has shown that the damaged mtDNA-encoded ATP6 gene will reduce ATP synthesis and increase ROS generation, which may contribute to VC progress (105). Based on the previously described role of ROS and MDF in VC, we have reason to believe that mitochondrial epigenetic abnormalities will promote VC.

Unlike nuclear DNA, mtDNA does not combine with histones, and DNA polymerase γ (Pol γ) is the only DNA polymerase encoded by the gene *POLG* that locals in mitochondria and takes charge of mtDNA replication and maintenance, leading to mitochondria having a relatively inefficient DNA repair system; as a result, mtDNA had low stability and was prone to mutation or deletion (106).

To determine whether mtDNA damage directly promotes AS, Yu et al. (86) constructed ApoE^−/−^ mice deficient for the mitochondrial polymerase-γ proofreading activity (polG^−/−^/ApoE^−/−^) model. They found that POLG^−/−^ApoE^−/−^ mice exhibited extensive mtDNA damage and oxidative phosphorylation defects but did not exhibit an increase in ROS. Moreover, POLG^−/−^ApoE^−/−^ mice had more severe AS and VC compared with POLG^+/+^ApoE^−/−^ mice; this indicates that POLG-related mtDNA damage could promote the development of AS and VC.

Another study reported that the mutation at position 5513 from G to A of mtDNA led to multiple mitochondrial respiratory chain enzyme defects, accompanying serving calcification in the basal ganglia (107) and the T-A nucleotide pair deletion in positions 3271–3273 of mtDNA led to calcification in patient's cerebrum (108). This suggests that mtDNA damage, mutation, and epigenetic alterations affect the VC process, but the specific mechanisms still require further study.

### Mitochondrial Dynamics

Mitochondria are highly dynamic and are constantly undergoing fusion, fission, transport, and degradation. As mitochondria take the central role in energy metabolism, mitochondrial dynamics and bioenergetics reciprocally influence each other and play a crucial role in VC (5, 109). Mitochondria regulate their morphology, quantity, distribution, and function through fusion and fission. This process is mediated by extensive protein machinery and the removal of damaged mitochondria by mitophagy. Each of these dynamic activities is necessary for the health of cells.

The mitochondrion is a double-membrane-bound organelle with a smooth outer membrane (MOM) and a highly folded inner membrane (MIM) that protrudes into the mitochondrial matrix compartment (cristae) (110). The MIM and MOM create the following two mitochondrial compartments: the matrix that is surrounded by the MIM and the inter-membrane space (IMS) between the MIM and MOM, which are partially connected *via* contact sites involved in cristae organization (111). When fusion is increased or fission is decreased, the mitochondria will form a tubular structure, while when fusion is decreased and fission is increased, the mitochondria will present a granular structure. Mitochondrial fusion and fission can be regulated by the highly conserved GTPase protein family, which completes mitochondrial inner and outer membrane remodeling through self-assembly and hydrolysis of GTP. Different from mitochondrial fusion, mitochondrial fission is the main mediated by a Drp1 protein (dynamin-related protein 1) with a molecular weight of 80 kDa located in the cytoplasmic matrix. The mitochondrial inner membrane is distributed with receptor proteins that can collect Drp1: mitochondrial fission factor (Mff), mitochondrial fission protein 1 (Fis1), and two mitochondrial elongation factors (MIEF1/MiD51 and MIEF2/MiD49). These proteins can collect Drp1 into the outer membrane of mitochondria and further form a spiral ring structure and then drive mitochondrial division through hydrolytic GTP. This process is active and dependent on energy (ATP), requires a sufficiently negative trans-MIM electrical potential, and is mediated by two mitofusins (Mfn1/Mfn2; MOM-fusion) and the optic atrophy 1 protein OPA1 (MIM-fusion).

Maintaining mitochondrial integrity and function requires regulating the equilibrium between mitochondrial fusion and fission *via* the balance of mitochondrial synthesis and degradation. The imbalance between mitochondrial fusion and fission causes apoptosis in several pathological processes, including VC (112). Likewise, mitochondrial fragmentation has been shown to be tightly linked with increased oxidative stress and mitochondrial depolarization, *via* differential modulation of mitochondrial fission–fusion proteins (113). Drp1 is a key regulator of mitochondrial fission. More importantly, Drp1 was enriched in calcified regions of human carotid arteries. Drp1 inhibition attenuated VC through attenuated oxidative stress (114). In addition, Drp1 knockdown has been shown to decrease the migration of VSMCs and attenuate VC *via* altering mitochondrial energetics and the levels of ROS (115).

Lactate exacerbated Drp1-mediated mitochondrial fission *via* the NR4A1/DNA-PKcs/p53 pathway, causing MDF. Moreover, lactate inhibits BNIP3-mediated mitophagy, eventually leading to increased apoptosis and promoted VC (116). Through inhibiting the expression and phosphorylation of Drp1, Quercetin alleviates Pi-induced oxidative stress and mitochondrial fission. Finally, *via* inhibiting VSMCs apoptosis attenuates VC (117).

Optic atrophy 1 (OPA1) is a critical regulator of mitochondrial fusion, and the AMPK/OPA1 pathway is linked to mitochondrial fusion and mitophagy (118). Through promoting mitochondrial fusion/mitophagy *via* the AMPK/OPA1 pathway, melatonin attenuates VC (119). Corresponding research shows that melatonin attenuates VC through inhibiting mitochondrial fission *via* activating AMPK and decreases the expression of Drp1 (120). Current studies have shown that in the process of VC, the expression of mitochondrial fission protein Drp1 is increased, while the expression of fusion protein OPA1 is decreased, and with the decrease of mitochondrial autophagy, damaged mitochondria cannot be effectively eliminated. By reversing these changes, *in vitro* and animal experiments have shown that VC can be alleviated.

### Oxidative Stress

Currently, it is well accepted that the optimal physiological range of ROS required to maintain vascular physiology. The major source of ROS comes from mitochondria: inner membrane complexes I, II, and III (121, 122) and the mitochondrial matrix and/or inner membrane-bound dehydrogenases (123, 124) and NADPH oxidases (NOXs) (125). While the majority of electrons flowing down the electron transport chain (ETC) redox gradient eventually reach complex V, 1%−3% of electrons react prematurely with oxygen in complexes I and III, forming superoxide and other ROS (80).

Physiologically, ROS helps maintain vascular function *via* regulating various redox-sensitive signaling pathways (126). Especially, VSMCs exhibit much higher mitochondrial proton leak during aerobic respiration than skeletal muscle fibers and cardiomyocytes. Increased proton leakage is often coupled with increased ROS generation, indicating that the mtROS may play a crucial role in VSMCs (127).

Redox homeostasis has been phrased as “the golden mean of healthy living” and plays a central role in life (128), which pervades practically all fundamental processes, from cytogenesis to death (129). Oxidative stress can be defined as an imbalance between the prooxidant-antioxidant balance in favor of the former, leading to a disruption of redox signaling, control, and/or molecular damage (130). However, at present, the term “oxidative stress” has been overstressed in some cases (131).

Oxidative stress is an important mediator of VC especially medial calcification (132). Excessive free radicals can cause oxidative stress, which activates apoptosis pathways, leading to the death of VSMCs and inducing VC (117, 133). H_2_O_2_ causes oxidative stress, upregulates Runx2 through Akt signal-dependent mode, further promotes a phenotypic switch of VSMC from contractile to osteogenic phenotype, and shows that Runx2 is necessary for oxidative stress-induced VSMC calcification (134). Another strong evidence that oxidative stress promotes VC is that *via* analysis of 159 asymptomatic men free of overt clinical atherosclerosis revealed increased NADPH oxidase (the NADPH oxidase family constitutes an important source of ROS)-mediated superoxide production associated with enhanced coronary artery calcium (CAC) (135). In animal levels, high-fat-diet-induced oxidative stress and VC were significantly inhibited in the Runx2-deficient mice (136). Moreover, in the CKD calcification model, VSMCs often exhibited mitochondria membrane potential abnormality and increased intracellular and mitochondria ROS, promoting the p65 nuclear translocation. Reducing ROS can inhibit p65 activation and reduce calcium deposition *in vitro* and *in vivo* (137).

Medial calcification is the main type of VC in patients with CKD, and hyperphosphatemia is a crucial risk factor for VC, especially in CKD (10, 138). Moreover, oxidative stress is known to positive correlation with declining renal function (139) Type III NaPi (PiT-1 and -2) transporter is proposed as the predominant route for cellular Pi uptake in VSMCs and essential for Pi-elicited osteogenic differentiation (140). High phosphorus causes Ca2^+^ overload mediated by PiT-1/2, leads to oxidative stress, activates the ERK-mTOR pathway, and is critical in Pi-induced VC (141). Peroxisome proliferator-activated receptor-γ coactivator-1α (PGC-1α) plays a key role in mitochondrial biogenesis and fatty acid oxidation (142). Overexpression of PGC-1α suppresses VC both *in vivo* and *in vitro* through reduced mtROS by enhancing sirtuin 3 expression (143).

Some antioxidants have an anti-VC effect. A study reported that MitoQ (a mitochondrial-targeted antioxidant) *via* activating the Keap1/Nrf2 pathway can enhance antioxidant capacity, reduce VSMC apoptosis, and ultimately attenuate VC (144). Fibulin-3 (another antioxidant) through inhibition of endogenous oxidative stress can attenuate phosphate-induced calcification of VSMCs (145).

### Mitophagy

Autophagy is an important means for the maintenance of intracellular homeostasis; mitophagy is a subtype of autophagy that is considered as a protective mechanism to clear the damaged or unnecessary mitochondria, and normal mitophagy also highlights a critical role in maintaining mitochondrial biogenesis (146, 147). Altered mitophagic flux by pharmacological or genetic maneuvers has been shown to influence the VC process (148). The main mechanism may be related to the reduction of ROS release and mitochondrial-related apoptosis. Since apoptotic bodies serve as nucleation sites for calcium phosphate precipitation (149), damaged mitochondria are the main source of ROS formation (150), and therefore, removing impaired mitochondria by mitophagy should occupy the central position. However, there are few studies on the role of mitophagy in VC, and its regulatory mechanism and function are still unclear.

It is currently known that mitophagy can alleviate VC by inhibiting phenotype transformation of VSMCs, and the possible mechanisms include the following: (1) mitophagy regulates mitochondrial biogenesis, when mitophagy is inhibited, the mass of mitochondria will be reduced significantly (reflected by mitochondrial DNA copy number), and consequently impaired mitochondrial biogenesis will promote VSMC apoptosis (92); (2) mitophagy helps to maintain the balance between oxidation and antioxidant system and ease oxidative stress, and BNIP3-mediated mitophagy can reduce mPTP opening rate, improve mitochondrial membrane potential, and restore mitochondrial function under ROS stress (116); (3) mitophagy contributes to maintain mitochondrial dynamics, and pathological mitochondrial fission impairs mtDNA that participates in ROS generation and mitochondrial structure (151). Excessive Drp-mediated mitochondrial fission induces ROS overproduction and evokes apoptosis (152); correspondingly, melatonin activates AMPK/OPA1 signaling and promotes mitochondrial fusion; and mitophagy reduces VSMC apoptosis, oxidative stress, and inflammation, and ultimately alleviates VC (119).

### Mitochondrial-Related Apoptotic

Autophagy inhibits apoptosis and necrosis by removing damaged mitochondria, and failure of autophagy can promote VC (153). The mitochondrial pathway is one of the main two apoptotic pathways [another: death receptor pathway] (154). The mitochondrial membrane integrity was disrupted in the calcified environment, resulting in the release of cytochrome C. Cytochrome C bound and activated apoptotic protease activating factor-1 (Apaf-1) as well as caspase-3 and caspase-9, causing DNA fragmentation and other changes that result in the occurrence of apoptosis (155, 156).

Gas6/Axl-PI3K/Akt is the classic pathway in VC, and akt activation leads to downstream signaling processes involving mitochondrial apoptosis regulators, such as Bcl2 (157). This was mediated by growth arrest-specific gene 6 (Gas6), which binds to Axl, the predominant receptor for Gas6, on the cell surface, and transduces the signal by Axl autophosphorylation (158). A study demonstrated that α-lipoic acid could alleviate VC by inhibiting the activation of the Gas6/Axl/Akt pathway, restoring mitochondrial function, and inhibiting mitochondrial-related apoptosis (157).

Pro-calcification factors such as high phosphorus lead to mitochondrial oxidative stress, which further induces VSMC apoptosis. In this process, PDK4 plays an important role, which is a critical mitochondrial matrix enzyme involved in cellular energy metabolism and may aggravate GP-induced oxidative stress and consequent VSMC apoptosis (159). Metformin, a well-known glucose-lowering drug, inhibits the PDK4/oxidative stress-mediated apoptosis pathway, therefore impairing the VSMCs' phenotypic shift to an osteogenic phenotype (92).

### Mitochondrial Energy Metabolism

Mitochondria provide almost 95% of the energy required for cell life activities (160). Its content and morphology are highly tunable to adapt to the energy requirements of different cells or activities (161). Mitochondria are key regulators of fatty acid oxidation, which plays a role in the development of calcification (162). Furthermore, fatty acid metabolism, in turn, regulates mitochondrial form, and function may also participate in the VC process (163).

Glycolysis is the main way for VSMCs to obtain energy and is responsible on average for ~70.1% of total ATP production (164, 165). Recent research indicates that the VSMCs' phenotypic flipping may be triggered by a metabolic transition (159). Aerobic glycolysis is a characteristic feature of VSMC osteogenic transdifferentiation (166). β-Glycerophosphate-treated VSMCs increased basal respiration, mitochondrial ATP production, and proton leak and had a more oxidative and less glycolytic phenotype, which may contribute to the onset of phenotypical trans-differentiation and calcification (165). In diabetes, glycation end products (AGEs) promote VC *via* excessive oxidative stress and tightly link with glucose metabolism. ROS increases the expression of pyruvate dehydrogenase kinase 4 (PDK4), which converts oxidation to aerobic glycolysis in VSMCs (159). Bone Gla protein (BGP), also known as osteocalcin (OCN), highly expresses in calcified tissues, shifts VSMCs toward the glycolytic breakdown of glucose, and stimulates VSMC calcification (167, 168).

**Figure 6:**
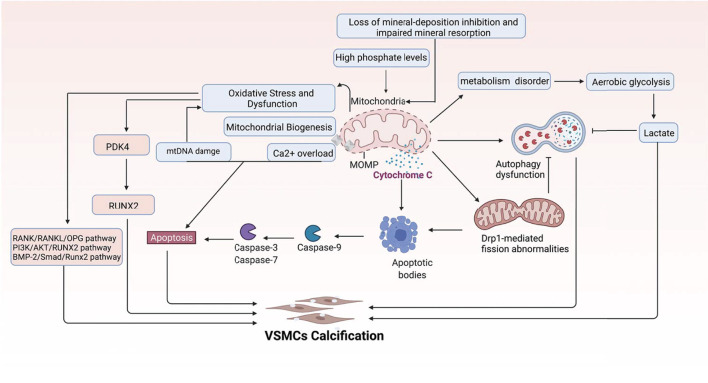
Multi-mechanistic pathways driving VC in mitochondria. This figure demonstrates the main mechanisms mediating VC centered on mitochondria.

Carnitine O-octanoyltransferase (CROT) is an enzyme involved in the transport of medium and long-chain acyl-CoA out of peroxisomes (169). The expression is significantly increased in calcified primary human coronary artery smooth muscle cells. Inhibition of CROT suppresses type 1 collagen secretion, restores mitochondrial proteome alterations, and suppresses mitochondrial fragmentation in calcifying VSMCs. Furthermore, CROT catalyzes the transfer of fatty acyl groups between CoA and carnitine. CROT siRNA increased the levels of selected fatty acids, including a VC inhibitor-eicosapentaenoic acid, and ultimately reduced the calcification of the aorta and carotid arteries without affecting bone density, liver, or plasma cholesterol and triglyceride concentrations (170, 171). In summary, mitochondria are one of the central organelles in VC, and many molecules and pathways are involved (Figure 6).

## Conclusion and Prospect

Vascular calcification (VC) is a complex biological process that significantly increases cardiovascular and all-cause mortality. However, the pathological mechanism of VC is still unclear, and the relationship between different periods of VC and disease development is still controversial (10). By far, many mechanisms involved in the regulation of VC have been gradually discovered, such as extracellular vesicles, non-coding RNA, and new forms of programmed cell death. Furthermore, advances in molecular imaging, material science, and big data technology, including multi-omics and network medicine, and the integration of these approaches help provide a more comprehensive map of VC. Several therapeutic measures have been shown to effectively inhibit VC in animal models and *in vitro* experiments. However, in clinical practice, there is still no specific therapy to reduce these calcifications and no evidence to support a better cardiovascular outcome with calcification reduction. Therefore, a more in-depth elucidation of the regulation mechanism and treatment progress of VC are crucial to the research of VC and the solution of clinical problems.

Moreover, the clinical-epidemiological features and lack of non-invasive specific diagnostic indicators, especially for microcalcification, are not well understood. VC especially coronary artery calcification (CAC) significantly increases the difficulty of percutaneous coronary intervention (PCI), and preevaluation of CAC lesions has a critical impact on subsequent treatment. Many imaging technologies have evolved in recent years to aid in the evaluation of calcified lesions, therefore, reducing the danger of intervention and improving the prognosis of surgery. These testing approaches have their own set of pros and cons, which are briefly summarized in Table 1. The diagnosis of microcalcifications mainly relies on optical coherence tomography (OCT) and 18FU-CT, both of which are invasive and expensive (172). Therefore, there is an urgent need to find methods and molecular markers for the non-invasive identification of early microcalcifications and to lay the foundation for the development of effective drugs for the treatment of or stabilization of microcalcifications.

**Table 1:** Comparison of different methods for the examination of calcified lesions.

**Imaging modalities**	**CT**	**Coronary angiography**	**IVUS**	**OCT**
Representative images	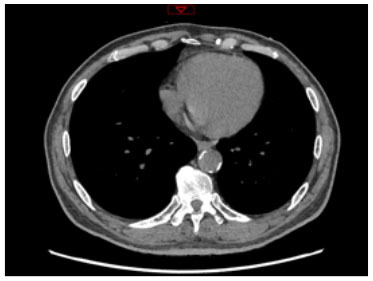	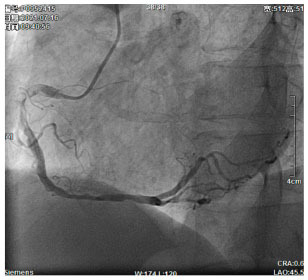	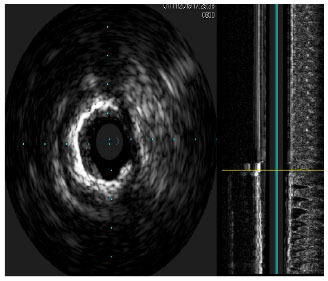	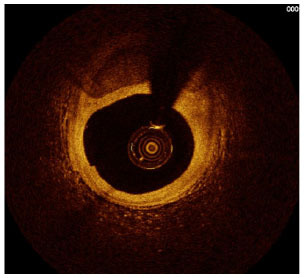
Invasive	No	Yes	Yes	Yes
Max resolution	0.4–0.6 mm	NA	100–200 μm	10–20 μm
Notable shortcomings	Inadequate spatial resolution for detecting microcalcifications	Low resolution	It cannot determine the depth of calcification.	Expensive and prevents visualization of deep calcium
Sensitivity	***	*	***	****
Location determination	***	*	***	****
Quantitative analysis	***	*	**	****

*~**** symbol indicates detection sensitivity/ability.

Mitochondria are highly specialized organelles in eukaryotic cells, which are important centers of energy metabolism and signal transduction. Imbalances in mitochondrial homeostasis, including fission, fusion, oxidative stress, calcium overload, mitophagy, and biogenesis, may trigger the onset and development of VC. There has been less clinical research on mitochondrial function and VC to date, although some *in vivo* investigations using animal models have revealed that certain medications or processes may participate in and influence the connection between mitochondrial homeostasis and VC (Table 2). However, many unanswered questions remain with regards to mitochondrial homeostasis in VC. Frist, the imbalances of mitochondrial homeostasis are systemic or cell-specific in VC. In other words, the influence weights are varied in different vascular cell types, just as the volume occupied by mitochondria in different cell types is diverse. Second, is mitochondrial homeostasis imbalance as a common phenomenon in VC or is it of different importance for different types of VC such as VC in chronic kidney disease and atherosclerotic VC? Third, does mitochondrial homeostasis play different roles in different stages of VC (microcalcification and macrocalcification)? Fourth, it is of interest to describe the interaction of different aspects in mitochondrial homeostasis such as oxidative stress and mitophagy.

**Table 2:** Therapy agents in vascular calcification target mitochondria.

**Agents**	**Pathway**	**Mito change**	**References**
Metformin	AMPK/PDK4/oxidative stress	Mitochondrial biogenesis (+)	(92)
		Function (+)	
		Mitophagy (+)	
		Apoptosis (–)	
Statin	Gas6/Axl-PI3K/Akt	Function (+)	(157, 158)
		Apoptosis (–)	
Fibulin-3	NOX4,CYBA/MMP2;MMP9/BAX/BLC2	Function (+)	(145)
		Oxidative stress (–)	
Mitoquinone	Keap1/Nrf2	Function (+)	(144)
		Oxidative stress (–)	
		Apoptosis (–)	
α-Lipoic acid	Gas6/Axl/Akt	Function (+)	(133)
		Apoptosis (–)	
Melatonin	AMPK/Drp1	Dynamics (+)	(119, 120)
	AMPK/OPA1	Function (+)	
Quercetin	Drp1	Function (+)	(117)
		Oxidative stress (–)	
		Dynamics (+)	
		Apoptosis (–)	

Finally, due to the lack of histone protection, mtDNA is more prone to damage after noxious stimuli, and thus, it is of great clinical value to further explore the molecular mechanism of preventing mtDNA damage. Moreover, it is noteworthy to point out that the epigenetic modification of nuclear DNA has been widely explored, but less is known about the mechanism of epigenetic modification of mtDNA and its role in vascular diseases, especially VC. It requires further investigation of the mechanism of mtDNA epigenetic modification and its role in vascular diseases, especially VC.

At the macro level, although there has been great progress in the study of VC, research is still few and flimsy compared with areas such as atherosclerosis. The results leading to this status may be related to the lack of recognized animal and cellular models. Additionally, extensive questions that need to be further explored by researchers are as follows. (1) Further studies are needed to better understand the drivers of osteogenic phenotypic transformation and, most importantly, to characterize the spatiotemporal regulation of these drivers. (2) Are there key regulators of the transition from microcalcification to macrocalcification? (3) How to choose a treatment for calcification: Inhibit the onset of calcification? Facilitate the transition from microcalcifications to macrocalcifications? (4) Currently, it is often assumed that phenotypic changes precede calcification, but this has not been conclusively demonstrated, and the VSMC phenotypic shift may be a cellular response to the osteogenic environment. (5) Is VC an adaptive protective mechanism against pathological damage suffered by the vasculature such as atherosclerosis? (6) Development of a non-invasive diagnostic strategy for vascular microcalcifications. (7) Establishing better and more stable animal and cellular calcification models. (8) Organs or cells cross talk involved in VC. (9) Angiogenesis and VC. (10) Vascular microenvironment and VC. The road ahead is long and hard. Persist, success is in the card.

## Author Contributions

ZL and QY searched and analyzed the literature. ZL wrote the manuscript. XY and JH reviewed and edited the manuscript. All authors have read and approved the final manuscript.

## Funding

JH Laboratory is supported by the National Natural Science Foundation of China (No. 81873651), Hunan Province Key R&D Project (2020SK2107), and Key Projects of Hunan Health and Family Planning Commission (20201901). ZL is also supported by the postgraduate Scientific Research Innovation Project of Hunan Province (CX20210917).

## Conflict of Interest

The authors declare that the research was conducted in the absence of any commercial or financial relationships that could be construed as a potential conflict of interest.

## Publisher's Note

All claims expressed in this article are solely those of the authors and do not necessarily represent those of their affiliated organizations, or those of the publisher, the editors and the reviewers. Any product that may be evaluated in this article, or claim that may be made by its manufacturer, is not guaranteed or endorsed by the publisher.
